# Hereditary Haemorrhagic Telangiectasia, an Inherited Vascular Disorder in Need of Improved Evidence-Based Pharmaceutical Interventions

**DOI:** 10.3390/genes12020174

**Published:** 2021-01-27

**Authors:** Ryan O. Snodgrass, Timothy J. A. Chico, Helen M. Arthur

**Affiliations:** 1 Department of Infection, Immunity & Cardiovascular Disease, Medical School, University of Sheffield, Sheffield S10 2RX, UK; rosnodgrass1@sheffield.ac.uk (R.O.S.); t.j.chico@sheffield.ac.uk (T.J.A.C.); 2 Biosciences Institute, Centre for Life, Newcastle University, Newcastle NE1 3BZ, UK

**Keywords:** BMP9/10, ENG, ACVRL1, VEGF, angiogenesis, arteriovenous malformation

## Abstract

Hereditary haemorrhagic telangiectasia (HHT) is characterised by arteriovenous malformations (AVMs). These vascular abnormalities form when arteries and veins directly connect, bypassing the local capillary system. Large AVMs may occur in the lungs, liver and brain, increasing the risk of morbidity and mortality. Smaller AVMs, known as telangiectases, are prevalent on the skin and mucosal lining of the nose, mouth and gastrointestinal tract and are prone to haemorrhage. HHT is primarily associated with a reduction in endoglin (ENG) or ACVRL1 activity due to loss-of-function mutations. ENG and ACVRL1 transmembrane receptors are expressed on endothelial cells (ECs) and bind to circulating ligands BMP9 and BMP10 with high affinity. Ligand binding to the receptor complex leads to activation of the SMAD1/5/8 signalling pathway to regulate downstream gene expression. Various genetic animal models demonstrate that disruption of this pathway in ECs results in AVMs. The vascular abnormalities underlying AVM formation result from abnormal EC responses to angiogenic and haemodynamic cues, and include increased proliferation, reduced migration against the direction of blood flow and an increased EC footprint. There is growing evidence that targeting VEGF signalling has beneficial outcomes in HHT patients and in animal models of this disease. The anti-VEGF inhibitor bevacizumab reduces epistaxis and has a normalising effect on high cardiac output in HHT patients with hepatic AVMs. Blocking VEGF signalling also reduces vascular malformations in mouse models of HHT1 and HHT2. However, VEGF signalling is complex and drives numerous downstream pathways, and it is not yet clear which pathway (or combination of pathways) is critical to target. This review will consider the recent evidence gained from HHT clinical and preclinical studies that are increasing our understanding of HHT pathobiology and informing therapeutic strategies.

## 1. Introduction

Hereditary Haemorrhagic Telangiectasia (HHT) is an inherited vascular disorder affecting up to 1 in 5000 people. It is an autosomal dominant disorder and the vast majority of patients (>85%) have inherited one allele encoding nonfunctional endoglin (*ENG*) or activin receptor-like kinase 1 (*ACVRL1*, also known as *ALK1*) allele [1]. A minority of HHT cases are due to mutations in *SMAD4* or *BMP9* (*GDF2*), but these have somewhat different clinical presentations. SMAD4 patients have a combined Juvenile Polyposis–HHT syndrome, whilst BMP9 patients display a mild HHT-like phenotype [2,3]. This review will focus on the two major patient groups, HHT1 and HHT2 that are caused by loss-of-function (LOF) mutations in *ENG* and *ACVRL1*, respectively.

HHT1 and HHT2 patients develop very similar clinical symptoms that result from sporadic vascular malformations, with clinical diagnosis based on the “Curacao criteria”. Patients with at least three of the following features are considered to have a definitive diagnosis of HHT: (1) multiple mucocutaneous telangiectases, (2) recurrent nosebleeds, (3) visceral organ arteriovenous malformations (AVMs) and (4) a first degree relative with HHT. Where possible, this diagnosis is confirmed by genetic testing. Telangiectases are small arteriovenous connections found on the skin and mucosal surfaces and are prone to bleeding in the nose and gastrointestinal (GI) tract. In fact, epistaxis from nasal telangiectases is the most highly penetrant phenotype, present in almost all young adults with HHT, and anaemia may be sufficiently severe to necessitate regular iron or blood transfusions. Larger AVMs may be present in the lung, liver and neural tissues. HHT1 and HHT2 patients differ in the incidence of affected tissues, with AVMs in the lung and brain more common in HHT1, whilst a spectrum of hepatic vascular malformations including AVMs are more common in HHT2 [4,5]. The reasons for these differences are not yet known.

## 2. BMP9/10 Signalling

**Figure 1:**
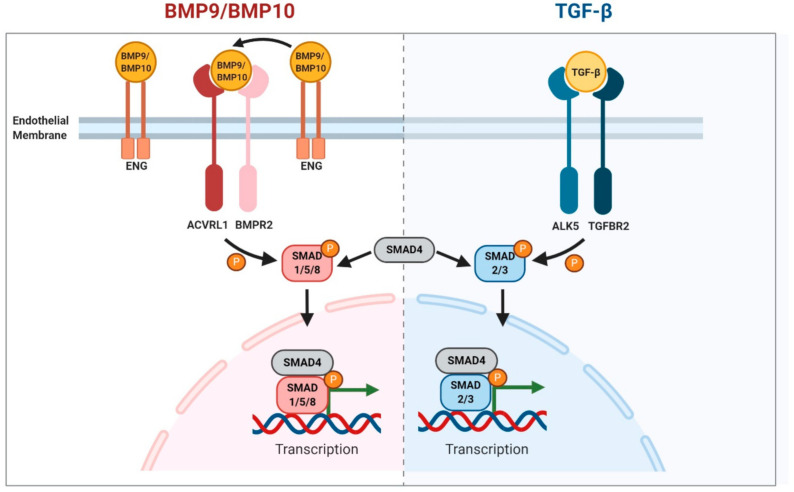
Schematic summary of BMP9/10 and TGFβ1 signalling pathways in endothelial cells. Figure created with BioRender (https://biorender.com/).

ENG and ACVRL1 proteins are expressed on the endothelial cell (EC) surface. They are also found in other cell types, for example, in fibroblasts, but overwhelming evidence from preclinical studies points to the critical nature of their *endothelial* role in protecting against vascular malformations in HHT [6]. LOF mutations in *ENG* and *ACVRL1* lead to disruption of bone morphogenetic protein 9/10 (BMP9/10) signalling. BMP9 (encoded by *GDF2*) and BMP10 are ligands of the TGFβ superfamily produced by the liver and right cardiac atrium, respectively, and released into the circulation in active forms [7,8,9,10]. ENG and ACVRL1 proteins have a high binding affinity for both BMP9 and BMP10 [11,12] that initiates signalling [13]. ENG has a large glycosylated extracellular domain that can form an ENG–BMP9/10 complex [14] and provides an EC surface reservoir of bound ligand [15]. To promote signalling, ENG forms a BMP9/ENG/ACVRL1 protein complex (or equivalent complex with BMP10 or potentially even a BMP9/10 dimer [16]). In this way, ENG acts as a coreceptor to promote BMP9/10 signalling through the signalling receptor kinase ACVRL1. ENG protein is released from the complex following recruitment of the type II receptor (either BMPR2 or ACTRIIB or ACTRIIA) which phosphorylates ACVRL1 kinase to activate and propagate the signalling cascade via activation of transcription factors SMAD1/5/8 (Figure 1). SMAD4 then associates with phospho-SMAD1/5/8 to form a SMAD transcription factor complex which shuttles to the nucleus to regulate genes that promote quiescence and migration. In parallel, TGFBR2/ALK5 stimulation following binding of TGFβ ligand results in phosphorylation of SMAD2/3. Phospho-SMAD2/3 then interacts with SMAD4 in order to move to the nucleus to regulate multiple genes including those that promote extracellular matrix secretion. Disruption of the BMP9/10 pathway leads to the endothelial cell defects seen in HHT and may be partly explained by a disrupted balance of BMP9/10 and TGF-β1 signalling pathways.

The critical importance of this pathway in HHT is confirmed by evidence from combined loss of BMP9 and BMP10 activities in mouse models that recapitulate the vascular malformations typical of HHT [17,18]. The requirement for loss of both ligands to generate AVMs pointed to functional redundancy. However, BMP10, but not BMP9, plays the critical role in maintaining normal vascular architecture in zebrafish [19,20]. Work in mice suggests some differences between BMP9 and BMP10 functions, where loss of BMP9 affects the lymphatic but not blood vasculature [21], and loss of BMP10 is embryonic lethal at E10.5 due to failure of heart development [8]. Replacing the murine BMP10 coding sequence with that of BMP9 partially rescues defects in heart development, but not sufficiently to survive to birth, pointing to an essential role for BMP10 in cardiogenesis [22]. Further investigations are required to interrogate the overlapping and different functions of BMP9 and BMP10 signalling in ECs in vivo, as they appear to be very similar in vitro [10]. Indeed, signalling outcomes may depend on type II receptor availability as BMP9 shows a preference for ACTRIIB over BMPR2, and low affinity for ACTRIIA, whilst BMP10 has similar binding affinity for all three type II receptors [23]. A better understanding of the roles of BMP9 and BMP10 in the vasculature would inform potential ligand treatment strategies to rescue clinical features of HHT1 (see below). Moving downstream of the signalling pathway, the critical importance of the canonical SMAD1/5/8 pathway in protecting ECs against vascular malformations is shown by the HHT-like phenotype in SMAD4 LOF mutation patients [2], and by the retinal AVMs that develop in neonatal mice with endothelial loss of either SMAD4 or SMAD1/5 [24,25].

Whilst considering the role of this pathway in HHT, it is important to note that there is some confusion in the literature regarding the critical ligands that protect against HHT, because TGFβ1 and 3 were originally thought to be the primary ligands for the ENG/ACVRL1 pathway. As discussed above, it is now clear that BMP9 and BMP10 have much higher affinities for ENG and ACVRL1 proteins than TGFβ1. It is even possible that rather than TGFβ1 signalling being reduced following depletion of ENG or ACVRL1 protein activity, TGFβ1 signalling may actually be increased due to an imbalance between BMP9/10 and TGFβ1 signalling in ECs (Figure 1).

## 3. Aetiology of HHT Disease

Although there is a growing consensus that HHT is caused by reduced BMP9/10 signalling in ECs, it is not yet clear why the vascular lesions are localised to specific organs and tissues. Nasal telangiectases and nosebleeds seem to be the most highly penetrant feature affecting over 90% of young HHT adults. Dermal and GI telangiectases accrue later in adult life, whilst pulmonary and cerebral AVMs may be present from birth, usually reaching their final size by adult life [26,27]. The first point to emphasise here is that the majority of the vascular architecture in HHT1/2 patients appears to be normal, supporting the conclusion that one wild type allele (for *ENG* or *ACVRL1*) is sufficient for development, maintenance and function of the vast majority of the vasculature. Therefore, to explain the focal nature of vascular lesions, a local second genetic hit has been postulated, and recent evidence has indeed confirmed biallelic loss of *ENG* or *ACVRL1* gene function in dermal telangiectases from HHT1 and HHT2 patients [28]. This mechanism is yet to be confirmed in larger AVMs from HHT patients when such tissue becomes available. However, this finding is entirely consistent with evidence from preclinical models that reproducibly develop AVMs when functional *Eng* or *Acvrl1* genes are deleted from ECs, but rarely when mice are heterozygous carriers of *Eng* or *Acvrl1* LOF mutations [6]. This second genetic hit event to generate *ENG* or *ACVRL1* null ECs will likely be a stochastic event that is entirely consistent with HHT vascular lesions that accrue with age such as dermal telangiectases.

If biallelic loss of *ENG* or *ACVRL1* drives the formation of sporadic vascular lesions in HHT, this only partly explains the phenotypic complexity of this disease. An explanation is also required as to the tissue location preference for AVMs and telangiectases. Considering the nasal telangiectases first, these lesions are the most highly penetrant defect in HHT1 and HHT2, and the nasal mucosal tissue is also an area of acute inflammation during frequent respiratory infections such as the common cold. Local inflammation would provide two additional stimuli that may be relevant to initiate telangiectasis formation. Firstly, inflammation triggers removal of the endothelial glycocalyx, and is associated with local release of inflammatory cytokines such as tumour necrosis factor-α (TNFα). This cytokine triggers events leading to cleavage of the extracellular domain of ENG protein, which is released as a soluble form into the circulation [29]. As HHT1 patients have baseline levels of 50% of the normal levels of ENG protein [30], then protein cleavage during inflammation may cause this to drop below the levels required to maintain the normal vascular architecture. It is interesting to note in this context that genetic variants of *ADAM17*, which encodes a major regulator of TNFα activity, are associated with pulmonary disease severity in HHT1 [31]. Secondly, inflammation resulting from infection or tissue injury generates a proangiogenic stimulus that has been shown in preclinical models to be required for AVM formation [6,32]. Organs that are frequently affected in HHT are all exposed to environmental and/or inflammatory insults. This is most obvious for the lung, skin, oronasal and GI tract. However, the liver is also exposed to blood draining from the gut which is rich in antigenic material including microbial debris. As a result, local inflammation is a normal part of liver homeostasis [33]. In addition, although cerebral tissue would normally be protected from environmental insults, there is an increased risk of embolic stroke and cerebral abscess caused by microthrombi passing through lung AVMs, even when these are small and clinically silent [26,27]. It therefore logically follows that there is an increased risk of proinflammatory microthrombi reaching the brain that may be insufficient to cause clinical stroke or abscess, but still provide sufficient pathophysiological proinflammatory trigger for vessel remodelling to form brain AVMs (BAVMs). Therefore, it may be no coincidence that lung and brain AVMs are both present in HHT1 patients more frequently than HHT2 patients. Furthermore, the variability of clinical symptoms even within the same HHT family carrying the same mutation (in *ENG* or *ACVRL1*) can be explained by this complex pathogenesis that depends on the timing of the second hit—a stochastic mutation event and/or exposure to an inflammatory or other environmental stimulus that promotes angiogenesis. Indeed, developmental angiogenesis may help drive the formation of congenital AVMs in the presence of biallelic *ENG* or *ACVRL1* LOF mutations. Ultimately, however, the precise reasons for the specific tissue distribution of vascular lesions in HHT remain to be uncovered in detail in future work.

Once an abnormal arteriovenous shunt is formed it becomes extremely challenging to reverse due to the increased blood flow. Added to this is evidence that loss of ACVRL1 signalling alters the endothelial response to shear stress [34,35]. Analysis of zebrafish embryos harbouring a LOF mutation in *acvrl1* showed increased EC numbers in cerebral arteries that further increased in response to blood flow to generate stabilised AVMs [35]. This increase in EC number to generate an AVM is not due to increased proliferation, but rather to reduced migration of ECs against blood flow leading to an accumulation of ECs that would normally have migrated towards the heart [36]. This reduced migration defect has also been confirmed in a mouse model of HHT1 [37]. In this way, due to a failure of normal EC migration against blood flow, congenital AVMs may further enlarge during developmental angiogenesis. Furthermore, additional local secondary events likely come into play as blood shunting via an AVM and bypassing a local capillary bed cannot efficiently exchange oxygen leading to local tissue hypoxia. Low oxygen leads to a local increased expression of many genes, including VEGF, which potentially drives a positive feedback scenario in HHT causing further angiogenic stimulation and AVM expansion.

Another important contributory element to AVM formation is the disruption of crosstalk between ECs and pericytes. Following loss of ENG, there is reduced pericyte–EC contact that may affect vasoregulation and vessel stability in HHT. Evidence from mouse models of HHT shows reduced vascular smooth muscle coverage and reduced pericyte–endothelial integration leading to vessel instability [38,39]. Reduced pericyte number and coverage have also been reported in sporadic brain AVMs (BAVMs) [40], and may also be the case in BAVMs in HHT patients.

To summarise, in addition to genetic loss of *ENG* or *ACVRL1*, developmental angiogenesis, blood flow and local environmental triggers driving neovessel formation, such as hypoxia and inflammation, are critical players driving the formation of AVMs in HHT.

## 4. Overlapping Endothelial Cell Abnormalities in HHT and Spontaneous BAVMs

Although up to 5% HHT patients develop BAVMs, the majority of BAVMs in patients presenting at neurology clinics are spontaneous. There is an inherent high risk of haemorrhage from a BAVM which accounts for the majority of haemorrhagic strokes in young adults. This risk has driven major efforts to better understand BAVM pathobiology, and in light of recent progress in this area it is important to consider whether there are overlaps between the downstream molecular changes that give rise to spontaneous and HHT BAVMs. Recent seminal work has revealed that somatic activating mutations in *KRAS* are associated with the majority of spontaneous BAVMs [41]. These constitutively active (CA) *KRAS* mutations are present in ECs of spontaneous BAVMs in a mosaic fashion. This parallels evidence from a mouse model of HHT1, where mosaicism for *Eng* mutations in ECs is sufficient to generate AVMs [37]. Thus, only a proportion of ECs need to harbour a new mutant allele to develop AVMs in both HHT and in spontaneous BAVMs. In HHT, the new mutation is a loss of the second functional *ENG* or *ACVRL1* allele, whereas in spontaneous brain AVMs it is gain of a CA-*KRAS* mutation. It is striking that ECs with gain of KRAS activity or LOF *ENG* mutations both show elevated phospho-ERK activity, suggesting that this may be a common pathway associated with AVM formation [42,43]. Furthermore, as inhibiting MEK signalling can reverse established AVMs due to activated KRAS in the zebrafish model [42], this finding may be relevant to HHT (as discussed below). However, it is not yet clear whether the proproliferative role of the activated RAS/RAF/MEK/ERK pathway makes a direct contribution to AVMs [42]. Similarly, it is not known to what extent loss of the antiproliferative role of BMP9 signalling in the absence of ENG or ACVRL1 proteins drives the increased EC proliferation seen in telangiectases from HHT2 patients and in AVMs in preclinical models of HHT [17,44,45,46,47]. Nevertheless, the similarities in cellular responses in AVMs in HHT and spontaneous BAVMs suggest that scientific progress in these two fields may be mutually informative [48].

## 5. Treatment Options for HHT

There are currently limited options for treating HHT. To date, the majority of therapeutic approaches in severe disease have focussed on invasive procedures to cauterise bleeding telangiectases or occlude phenotypic AVMs (where accessible) to reduce disease symptoms or risk of complications such as stroke. Depending on the location of symptomatic vascular lesions and severity of symptoms, the feeding artery of an AVM can be physically occluded to prevent arteriovenous shunting of blood. This is frequently achieved by implantation of intravascular metal coils to occlude pulmonary AVMs to successfully restore blood oxygenation levels. However, access to symptomatic AVMs in the brain and liver is more challenging and presents a higher risk. Importantly, embolization of asymptomatic AVMs is not recommended in HHT. Liver transplant is recommended where hepatic VMs are sufficiently severe to cause high output heart failure. The advances in understanding the molecular and cellular defects that drive HHT and the availability of reproducible preclinical models have driven further investigations to identify optimal pharmaceutical strategies to prevent or reverse established AVMs. Translation of these fundamental findings to the clinic ultimately depends on the availability of suitable safe drugs. These research efforts are summarised below and in Table 1.

### 5.1. Increasing Expression of ENG or ACVRL1 Genes

Using a myoblast cell line stably expressing an *Id1* reporter (C2C12BRA) to screen 700 FDA-approved drugs, Tacrolimus (also known as FK506 or Fujimycin) was identified as a potent activator of the BMP9-ACVRL1-BMPR2-SMAD1/5/8 signalling cascade [59]. This drug is widely used clinically as an immunosuppressant, for example, in organ transplantion, but has also been shown to increase *ENG* and *ACVRL1* expression [60]. Tacrolimus is also suggested to be useful in the treatment of pulmonary arterial hypertension (PAH), a disease associated with genetic defects in BMP signalling. Three end stage PAH patients all showed stabilized cardiac function and an increase in BMPR2 expression following Tacrolimus treatment [61]. A case report shows clinical improvements following Tacrolimus therapy in an *ACVRL1* patient with a combined HHT PAH syndrome [62]. However, a recent randomised trial showed only minor effects of topical Tacrolimus nasal treatment on epistaxis [51] and further work is needed to evaluate this therapy in HHT.

Recent work has shown that ectopic expression of human/mouse ACVRL1 in mouse models of HHT1 and HHT2 can prevent AVMs [63]. Although this finding is in line with the recessive nature of LOF *ENG* and *ACVRL1* mutations in the presence of exogenous ACVRL1 expression, this type of approach is difficult to apply clinically. If Tacrolimus increases expression of the unaffected allele in HHT patients, this has the potential to be beneficial, but only if the vascular lesions in HHT are caused by haploinsufficiency. This may be true if a localised bout of inflammation led to a temporary loss of ENG or ACVRL1 protein, but if the majority of lesions arise from biallelic LOF mutations (due to the genetic two hit mechanism), this strategy would not be useful. Increased understanding of disease mechanisms in HHT patients is clearly essential and awaits genetic analysis of larger AVMs, as discussed above.

Patient-specific mutant allele correction using a CRISPR/Cas9 approach is possible in principle but is currently too high risk due to risk of uncontrolled off-target effects. In terms of a personalised gene therapy approach, patient-specific exon skipping has moved to clinical trials in genetic diseases such as Duchenne muscular dystrophy (DMD). This uses a therapeutic antisense oligonucleotide that allows the transcriptional machinery to bypass pathogenic DMD mutations in specific exons (amenable to exon skipping) to produce a slightly shorter but still partially functional dystrophin protein [64]. This approach is less likely to be useful for *ACVRL1* and *ENG*, where there are no known redundant protein regions. However, treatments enabling stop codon read-through [65] may be applicable to specific HHT1 and HHT2 patients where the disease-causing mutation is a premature stop codon in the *ENG* or *ACVRL1* coding region. This type of mutation leads to premature termination of translation and a truncated nonfunctional ENG or ACVRL1 protein. Although its efficacy is highly variable, stop codon readthrough is now under evaluation in clinical trials for some cystic fibrosis patients and may offer hope to those HHT patients with disease-causing nonsense mutations.

### 5.2. Increasing BMP9/10 Ligand Availability

As discussed above, HHT1 is likely due to a reduced level of BMP9/10 signalling through depleted ENG protein levels. Therefore, it may be possible to bypass the requirement for ENG and increase BMP9/10 signalling through ACVRL1 kinase by increasing the amount of circulating BMP9/10 ligand. Clearly, this approach would be ineffective to bypass local loss of ACVRL1 signalling kinase activity in HHT2. However, a major potential problem using exogenous BMP9/10 is the propensity of both these ligands to drive heterotrophic bone formation [10]. Some exciting progress has been made in reducing the osteogenic properties of BMP9 whilst maintaining its EC signalling properties [10] and potentially similar changes could be applied to BMP10, which may have a higher protective effect in HHT [20].

### 5.3. Targeting Proangiogenic Growth Factor Signalling

**Figure 2:**
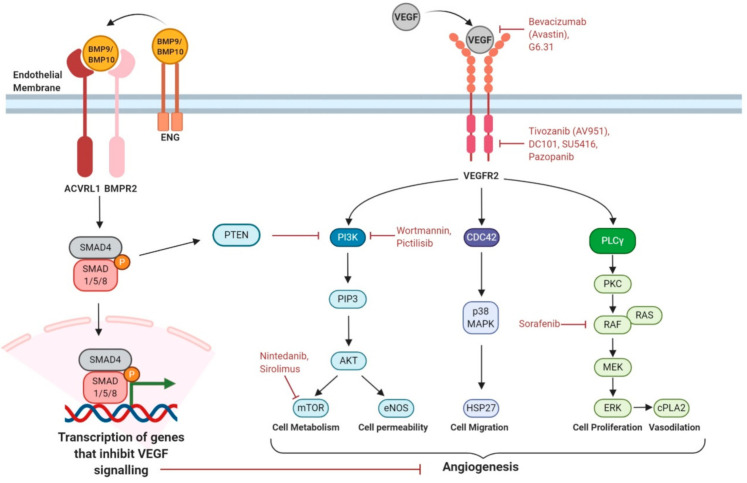
Schematic summary of crosstalk between BMP9/10 and VEGF signalling pathways in endothelial cells. Figure created with BioRender (https://biorender.com/).

VEGF(A) signals through VEGFR2 receptor to activate a range of downstream pathways regulating cell metabolism, proliferation, survival and permeability (Figure 2). BMP9/10 signalling affects the endothelial cell’s response to VEGF stimulation in at least two ways. First, BMP9/10 signalling prevents inactivation of PTEN, consequently inhibiting PI3K activity and reducing AKT activation responses. Second, downstream BMP9/10 signalling inhibits VEGF signalling outcomes in ways that remain to be defined but require a minimum of two hours, implying requirement for downstream gene expression [44]. In consequence, loss of BMP9/10 signalling leads to an untempered response of VEGF such as increased endothelial cell proliferation, which contributes to AVM formation. Drugs targeting VEGFA/VEGFR2 signalling and downstream mediators that have shown benefit in HHT (patients and/or preclinical models) are indicated in Figure 2 and summarised in the table.

A marked improvement of HHT disease symptoms was noted when the anti-VEGF antibody Bevacizumab was used to treat cancer in an HHT patient [66]. This initial serendipitous finding led to the realisation that reducing VEGF activity was beneficial in HHT and was followed up by successful (nonrandomised) clinical trials showing improved cardiac function in HHT patients with hepatic VMs and reduced epistaxis [49,50]. A major caveat is that to be effective intravenous Bevacizumab is repeated over a substantial period and can incur significant side effects including hypertension, thrombosis and impaired healing. Targeting VEGF signalling has also been shown to be effective in protecting against AVM formation in many animal models of HHT [37,43,55]. However, VEGF signalling is complex and drives numerous downstream pathways, and it is not yet clear which pathway (or combination of pathways) is critical to target. Furthermore, there is limited understanding to date of the molecular interaction between VEGF signalling and BMP9/10 signalling, or how this is disrupted in HHT. Advances in this area are essential to optimise and improve therapies targeting VEGF signalling.

Multisite phosphorylation of the VEGFR2 receptor following VEGF ligand binding leads to activation of various signalling pathways that regulate EC proliferation, migration and adhesion, all of which are required to regulate normal angiogenic responses. These downstream pathways include the phospholipase Cγ (PLCγ)–ERK1/2 pathway, the PI3K–AKT–mTOR pathway and CDC42-p38 pathway (Figure 2). Although BMP9 is known to counter the proangiogenic effects of VEGF and act as a circulating vascular quiescence factor [12,67], it is not known how exactly this is achieved by crosstalk between BMP9/10 and VEGF signalling pathways. There may simply be increased circulating levels of VEGF in HHT patients [68], perhaps due to local tissue hypoxia (as discussed above) or to an altered balance of ALK5/ACVRL1 signalling [69] and it is this increased VEGF that disturbs the balance of VEGF signalling to generate AVMs. However, this systemic increase in VEGF would not in itself explain the localised nature of AVMs in HHT.

EC-based studies in vitro have been used to interrogate the molecular interactions between BMP9/10 and VEGF signalling. BMP9 inhibits PI3K signalling by increasing activity of PTEN, a phosphatase that decreases PI3K activity [44]. In this way, localised loss of BMP9 signalling would drive a corresponding local increase in PI3K–AKT–mTOR signalling (Figure 2). Increased activation of PI3K is also seen when ACVRL1 levels are reduced either in HHT2 patients or mouse models of HHT2 and targeting PI3K can protect against AVM formation in the HHT2 neonatal mouse retina [17]. Important considerations limiting translatability of these findings are uncertainty over which PI3K isoforms are involved and clinically significant adverse effects of first generation PI3K inhibitors [70].

ECs lacking ENG protein show an increased rate of recycling of VEGFR2 to the plasma membrane which may contribute to enhanced downstream VEGF signalling [37]. Intriguingly, *ENG*-depleted ECs show reduced VEGFR2 expression compared with wild type cells, as well as reduced VEGFR2 phosphorylation in response to VEGF, yet still show increased ERK and AKT activation downstream of VEGF stimulation [43,71]. An added complexity is that a minimum of 2 h preincubation of cells with BMP9 can block VEGF stimulated cell responses, suggesting there are yet unidentified downstream factors involved in crosstalk between these two pathways [44]. These interactions are summarised in Figure 2.

As targeting VEGF with Bevacizumab requires intravenous delivery, some investigations have focussed on orally effective receptor tyrosine kinase inhibitors (TKI) used in oncology. These often target a broad range of growth factor receptors, and because HHT studies have recruited either few or single cases it is too early to conclude whether these provide a useful approach. The largest of these studies in seven HHT patients suggested that the TKI Pazopanib gave some benefit in reducing epistaxis [52]. Ongoing larger trials should provide firmer evidence in this area. In a preclinical model of HHT2, none of the 4 TKI drugs tested reduced wound-induced AVMs, but Sorafenib and a Pazopanib analogue (GW771806) did significantly improved gastrointestinal bleeding [56]. An interesting recent study has pointed to the power of a combination drug approach [54]. In mice with HHT (due to antibody blockade of BMP9/10) with the receptor TKI, nintedanib, together with the mTOR inhibitor, sirolimus, prevented as well as reversed retinal AVMs. This same drug combination reduced gastrointestinal bleeding and anaemia in an independent mouse model of HHT (inducible *Acvrl1*-deficient adult mice) [54].

In addition, VEGFR1 (also known as FLT1) acts as a natural decoy of VEGF signalling, trapping the VEGF ligand away from the VEGFR2 receptor. HHT2 mice show downregulation of VEGFR1 protein leading to enhanced VEGF signalling that will inevitably contribute to abnormal endothelial responses [46,72].

### 5.4. β-Blockers

Arteriovenous shunts are high flow abnormalities that deliver blood at high pressure into weak walled veins designed for low pressure flow. The small arteriovenous shunts in mucosal telangiectases are at high risk of rupture, likely on the venous side. Thus, blood pressure lowering medication may be beneficial. Propranolol, a nonselective β-blocker with antiangiogenic effects, has shown some benefit used topically on nasal telangiectases [73], although a double blind randomized placebo-controlled trial using topical application of the β-blocker Timolol showed no benefit on epistaxis [53].

### 5.5. Enhancing Pericyte–Endothelial Cell Interactions

Although Thalidomide was temporarily taken off the market following the tragic teratogenic outcomes when used to treat nausea in pregnant mothers in the 1960s, it has recently shown promise as a treatment for epistaxis in HHT [38,74]. Thalidomide has antiangiogenic effects by increasing EC–pericyte interactions. These cell–cell interactions are disrupted following endothelial loss of *ENG* or *ACVRL1* and Thalidomide appears to compensate for this to increase vessel stability. Mechanistic understanding of this effect is based on findings that loss of *Eng* in ECs leads to reduced crosstalk to adjacent muscle cells, leading to reduced downstream paracrine TGFβ signalling [75]. The resultant reduction in vascular mural cell coverage in mouse models of HHT are rescued following increased *PdgfB* expression stimulated by thalidomide [38,57]. However, in addition to its teratogenic effects, thalidomide can induce irreversible neuropathy so less neurotoxic derivatives are now under investigation.

The angiopoietin–Tie2 pathway is also critical for maintaining vessel stability. Angiopoietin-1 (ANGPT1), produced by mural cells, activates the endothelial TIE2 receptor to maintain vascular smooth muscle cell (VSMC) coverage in mature blood vessels. Following an inflammatory stimulus, ANGPT2 is rapidly released by ECs and inhibits ANGPT1-mediated TIE2 activation, leading to destabilisation of the VSMC–endothelial interaction and pericyte detachment. Interestingly, endothelial loss of *Smad4* in mouse leads to increased *Angptl2* and downregulated *Tie2* expression. The endothelial-specific *Smad4* null mouse model of HHT has an increased EC footprint and retinal AVM formation that could be prevented by inhibiting ANGPT2 function with LC10 [58]. However, the link to HHT remains to be clarified as circulating levels of ANGPT2 have been reported as reduced in HHT patients [76].

## 6. Summary and Conclusions

Recent advances in understanding the biological basis of HHT and the development of a range of preclinical models for drug testing has led to a gear change in efforts to find effective treatments for HHT. Patient-specific therapies/personalised medicine options may be required depending on individual disease symptoms and mutations. If similar cellular and molecular changes cause both small telangiectases and larger AVMs, with just a difference in scale, it is also possible that treatments targeting epistaxis may be equally effective for attenuating AVM progression in major organs. Certainly, VEGF and its downstream pathways are a major focus of current investigations along these lines. Pharmaceutical treatment that could reverse the vessel remodelling in AVMs would be life changing for HHT patients with severe morbidities. Finally, with better understanding of the molecular and cellular defects in HHT lesions, there is real hope that effective therapies are on the horizon.

## Figures and Tables

**Table 1:** Pharmaceutical rescue of Hereditary haemorrhagic telangiectasia (HHT)-related phenotypes in patients and preclinical models.

Drug	Target	Number of HHT Patients	Outcomes	Reference
Bevacizumab (Avastin)	Anti-VEGFA antibody	24	Reduced epistaxis and improved cardiac function in liver VM patients with HOHF	[49]
Bevacizumab (Avastin)	Anti-VEGFA antibody	238	Reduced epistaxis	[50]
Tacrolimus (FK506)	Increased activation ACVRL1	24 (+24 placebo)	Reduced epistaxis	[51]
Pazopanib	TKI	7	Some improvement in Hb and epistaxis	[52]
Timolol	β-adrenergic blocking agent	28 (+28 placebo)	No change in epistaxis	[53]
Thalidomide	Increased PDGFB expression	7	Reduced epistaxis	[38]
**Drug**	**Target**	**Mouse Model**	**Outcomes**	**Reference**
Wortmannin, Pictilisib	PI3K inhibitor	*Acvrl1-iKOe* and *Eng-iKOe* neonates	Reduced retinal AVMs	[17,37]
Nintedanib and Sirolimus	TKI and mTOR inhibitor	Neonatal antibody blockade of BMP9/10	Combination therapy reduced and reversed retinal AVMs	[54]
DC101	Anti-VEGFR2 antibody	*Eng-iKOe* adult	Prevents AVMs and HOHF	[43]
G6.31	Anti-VEGFA antibody	*Acvrl1-iKOe* adult	Prevention of wound induced dermal AVMs; possible reversal of established wound AVMs	[55]
Sorafenib and Pazopanib analogue (GW771806)	TKI	*Acvrl1-iKOe* adult	Each drug alone significantly improved Hb and GI bleeding but did not prevent wound-induced skin AVMs.	[56]
SU5416	VEGFR2 inhibitor	*Eng-iKOe* neonate	Significant reduction in retinal AVM size	[37]
Thalidomide	Increased PDGFB expression	*Eng+/−* and *Acvrl1-iKO* adult	Increased SM coverage of dermal and cerebral vessels, reduced cerebral haemorrhage	[38,57]
LC10	ANGPT2 inhibitor	*Smad4-iKOe* neonate	Prevents retinal AVMs	[58]

Abbreviations: GI, gastrointestinal; Hb, haemoglobin; HOHF, high output heart failure; -iKO(e), inducible gene knockout (Endothelial Cell (EC)-specific); TKI, tyrosine kinase inhibitor. VM: vascular malformation; AVM: arteriovenus malformation.

## Data Availability

Data sharing not applicable.
